# Antimicrobial resistance: The complex challenge of measurement to inform policy and the public

**DOI:** 10.1371/journal.pmed.1002378

**Published:** 2017-08-17

**Authors:** Didier Wernli, Peter S. Jørgensen, Stephan Harbarth, Scott P. Carroll, Ramanan Laxminarayan, Nicolas Levrat, John-Arne Røttingen, Didier Pittet

**Affiliations:** 1 Global Studies Institute, University of Geneva, Geneva, Switzerland; 2 Global Economic Dynamics and the Biosphere, Royal Swedish Academy of Sciences, Stockholm, Sweden; 3 Stockholm Resilience Centre, Stockholm University, Stockholm, Sweden; 4 Infection Control Program, University of Geneva Hospitals and Faculty of Medicine, Geneva, Switzerland; 5 WHO Collaborating Centre on Patient Safety, University of Geneva Hospitals and Faculty of Medicine, Geneva, Switzerland; 6 Department of Entomology and Nematology, University of California, Davis, Davis, California, United States of America; 7 Institute for Contemporary Evolution, Davis, California, United States of America; 8 Princeton Environmental Institute, Princeton, New Jersey, United States of America; 9 Center for Disease Dynamics, Economics & Policy, Washington, DC, United States of America; 10 Faculty of Law, University of Geneva, Geneva, Switzerland; 11 Department of Global Health & Population, Harvard T.H. Chan School of Public Health, Harvard University, Boston, Massachusetts, United States of America; 12 Norwegian Institute of Public Health and University of Oslo, Oslo, Norway

## Abstract

Didier Wernli and colleagues discuss the role of monitoring in countering antimicrobial resistance.

**Summary points**

- Antimicrobial resistance (AMR) is developing in many pathogenic bacteria, threatening to compromise the effectiveness of crucial medical treatments.
- Member States of the United Nations (UN) have reiterated their commitment to tackle AMR at the UN General Assembly held in New York City on 21 September 2016. The main challenge is now implementation of the Global Action Plan (GAP) adopted by the World Health Organization (WHO) in 2015.
- There are currently large information gaps about the global governance of AMR regarding both the magnitude of the problem and national responses.
- Expanding national and subnational monitoring by integrating measurements ranging from assessments of drivers of AMR to responses can increase political buy-in, societal participation, and implementation of agreed policies.
- WHO should lead the way to expand monitoring of progress regarding AMR control, but a broad coalition of global health actors is needed to build a robust approach in a significant number of countries.

Antimicrobial resistance (AMR) is increasing in a wide range of pathogens, causing morbidity and mortality globally, and threatening modern medicine. While the long-term impact of AMR on human societies remains uncertain [1], the conservation of antimicrobials’ effectiveness has become an urgent priority. Tackling this ubiquitous problem requires coordination among countries and across sectors that include human and animal health, the environment, development, and trade. Previous attempts at orchestrating such a response have been insufficient, but growing concern about AMR culminated in the adoption of a Global Action Plan (GAP) by the World Health Organization (WHO) in 2015 [2], followed by a political declaration at the General Assembly of the UN in 2016 [3]. Both documents recognize AMR as an interlinked biological and social problem driven by rising world population, exacerbated by the misuse of antimicrobials in human and animal health, compounded by globalization, and made more pressing by the lack of development of new drugs. In many developing countries, a high burden of infectious diseases, rising consumption in human and animal health, limited access to quality medicine, and poor public health infrastructure create conditions for the problem to worsen [4]. Governments, which have the ultimate responsibility to tackle the problem, have now started to deliver their national strategies based on the GAP [5]. To support implementation of the GAP, we see an immediate need to expand monitoring of countries’ commitments through an integrated approach to measure AMR.

## Current situation

Progress has been accomplished regarding AMR monitoring. First, knowledge about the causes, consequences, and magnitude of the problem has improved through research and better surveillance [6]. Second, the amount and quality of relevant data on AMR from local, national, or regional centers for disease control have been enhanced. Third, we understand better what needs to be measured and how to do it. A generic set of national performance indicators has been proposed [7], and WHO, which recently released a list of pathogens for which new antimicrobials are urgently needed [8], has also suggested indicators for monitoring implementation of the GAP. Additional relevant metrics include the drug resistance index, which aggregates data on bacterial resistance to multiple antibiotics and provides a useful measure of the severity of AMR [9]; and the defined daily dose per capita per year, which makes antimicrobial consumption comparable across countries [10].

However, major information gaps in the global governance of AMR—i.e., how we attempt to limit AMR globally—remain. First, surveillance data are lacking for low- and middle-income countries (LMICs). There are also more epidemiological data than WHO has access to, particularly from private hospitals and laboratories, which limits international reporting [6]. In 2014, for example, WHO reported data for carbapenem resistance in *Klebsiella pneumoniae* for only 71 countries out of 194 [11]. Second, variety in methodology and data collection hampers comparability across regions. Third, our understanding of the overall clinical and public health burden of AMR is limited. Fourth, few data are available on the state of national responses by countries. Finally, more needs to be done to integrate measurements on the multiple dimensions of the problem, as it is currently difficult to say whether AMR requires more attention in Italy or Canada, in Mexico or Thailand.

## Rationale

Addressing these information gaps is critical for successful implementation of the GAP. First, harmonized data collection will provide useful measures for individual countries to benchmark their national and subnational performance against others and help them tackle the problem based on the best scientific evidence. This could stimulate both domestic and transnational policy debates and help AMR remain on the political agenda. Second, as for many problems of international cooperation in which countries can free ride on the efforts of others, improved monitoring will reduce information uncertainty about the current situation and responses and create better conditions for cooperation through joint effort to produce global public goods [12]. This is extremely important because the increasing global movement of people and goods means that the preservation of antibiotic susceptibility depends largely on the magnitude of the weakest national efforts. Third, regular data collection across different countries could serve as a basis for a longitudinal assessment of AMR control and contribute to improve our understanding of where, when, how, and why particular interventions work, guiding the implementation of national and subnational policies. Fourth, better monitoring may result in greater involvement of and advocacy from health professionals, patients, and consumers, whose participation is critical to tackle AMR. Ultimately, the goal of expanded monitoring is to trigger a virtuous circle in which the collection of data constantly improves our understanding and capacity to tackle AMR, which in turn calls for new evidence.

## How to expand monitoring?

Because AMR control is complex, the goal should be to measure the problem and progress across its multiple dimensions while at the same time making it more tractable to policy makers and the public. Over the last decade, several instruments aimed at measuring performance in the economic, education, environmental, and health sectors have been developed [13]. Lessons learnt from their implementation—including concerns about the validity of composite indexes, which seek to summarize many dimensions in one number—call for a robust approach to ensure their effectiveness [14,15]. From the more general to the more specific, 5 design challenges are important to expand monitoring of AMR control.

The first is to choose an appropriate scientific approach to conceptualize AMR and related control efforts. Drawing from the growing literature on AMR determinants and interventions [7,16,17], a social-ecological approach that corresponds to “an integrated perspective of humans-in-nature” [18] could be adapted. The Driver-Pressure-State-Impact-Response (DPSIR) framework, which has been used by the European Environment Agency to respond to multifactorial problems, has been highly instrumental in developing pragmatic system-wide indicators in conservation biology [19]. Such a framework seems particularly appropriate to address AMR, as it integrates many variables from both the ecological and social dimensions of the problem.

The second challenge is to define what to measure. As there are different levels of economic development and multiple models of organization for the health, social, and political systems, it is critical to recognize that intrinsic diversity is an important parameter. Even ecological determinants of AMR might differ significantly across countries because of climate and other causes. Given this diversity among and within countries, AMR monitoring should be based on 3 main components: (1) the epidemiological situation and its impact on human health and societies (outcomes); (2) current drivers and practices (process), including antibiotic over-, mis-, or underuse; and (3) regulations and control policies (structures) [20]. Assessing performances within the 3 components for every country will help to identify different patterns of countries, which might be useful for screening countries at risk—for example, when a country has a weak regulatory framework, increasing antibiotic use, and a growing burden of AMR.

The third challenge is to select appropriate measurements from human, animal, and planetary health, as illustrated in Tables 1–3. Potential measurements that reflect the multidimensional nature of the problem are characterized in Table 1. Following the description above, the first column is organized around process, outcomes, and structures, and the second column further divides these components into the 5 categories of the DPSIR framework. The result is that measurements of process encompass both the primary “driving forces” in the use of antibiotics, such as the burden of infectious diseases or access to sanitation, and “pressures” that characterize the use of antibiotics both quantitatively and qualitatively. Measurements of outcomes can be divided into the current “state” of the problem in terms of the epidemiology of the most significant pathogens in human health and their “impact,” such as mortality and morbidity (potential measurements of state and impact are further detailed in Tables 2 and 3, respectively). Finally, measurements of structures are about the “responses,” which include the adoption of a national action plan and the enactment of regulations in human medicine and agriculture. The existence of regulations is not sufficient, as enactment does not equal enforcement. Measurements of implementation such as awareness campaigns, antibiotic stewardship programs, or surveillance capacities are critical for this component. In addition, over-the-counter availability of antibiotics can provide information about the level of enforcement. Importantly, given the delays between enactment and the effects of policies to tackle AMR, the proposed framework includes metrics appropriate to different points in the development of AMR control programs—from the early stages of the process (e.g., changes in law, knowledge, attitudes, and norms) to the outcomes (e.g., modifications of key health indicators such as the rate of multidrug-resistant bloodstream infections) [21].

**Table 1:** Candidate measurements to be considered as part of monitoring AMR and its control.

Component	DPSIR framework	Measurements	Rationale	Data source and feasibility
**Process:** drivers, knowledge, norms, and behavior	Driving forces: determine the human need for antibiotics	Burden of infectious diseases in human health	The burden of infectious diseases drives the use of antibiotics in the first place	Data on infectious disease burden are compiled by WHO and IHME
		Access to sanitation, safe drinking water, and waste water treatment	Sanitation regulates transmission	Data on the status of sanitation facilities are available from WHO/UNICEF
		Consumption of meat products	Intensive farming drives antibiotic use in agriculture	Data compiled by FAO
	Pressures: Both quantity and quality of antibiotic use exert pressure (over- and misuse of antibiotics)	Overall consumption of antibiotics in human and animal health	Estimate of selection pressure behind the correlation between use and resistance	Global estimates in the literature [22,23]
		Nonprescription availability (over the counter)	Nonprescription availability and misuse (proxy for strength of the regulatory framework)	Can be measured through testing (systematic review conducted in 2011) [24]
		Awareness of AMR among public and health professionals	Lack of awareness drives misuse of antibiotics	Various data from literature; WHO multicountry awareness study in 2015
		Access to quality antimicrobials in human health	Access to quality antimicrobials reduces misuse in human health	The proportion of the population with access to affordable, essential drugs on a sustainable basis is computed by the UN
		Appropriate use of antibiotics in hospitals and community	Inappropriate use of antibiotics is a driver of resistance. Adherence to best practices in terms of use can reduce the overall consumption of antibiotics	Some data in the literature (US CDC and ECDC)
**Outcomes:** current situation and burden of AMR	State: AMR epidemiology	Prevalence of most important resistant pathogens in hospitals, the community, and agriculture	Measure of the magnitude of the problem	Various data are collected at the national and regional level; WHO report on surveillance
	Impact on human health and societies	Human health burden of AMR from important pathogens for public health (morbidity and mortality)	Measure of the direct health consequences of AMR	Estimates from literature and national centers for disease control
		Economic burden of AMR	Current impact of AMR as an economic cost for society	Estimates from literature
**Structure:** policy and regulatory strategy framework	Responses in management tactics	Adoption of a national action plan based on the WHO global action plan	Measure of countries’ basic commitment to tackle AMR	Up-to-date database available from WHO
		Implementation of infection prevention and control	Infection prevention and control reduces spread of AMR pathogens, limits the AMR reservoir, and cuts antibiotic use	WHO country situation analysis [25] and data available in the literature
		Regulation of agriculture to limit nontherapeutic use	Agricultural use drives resistance via the physical environment and food chain	WHO country situation analysis; data in literature
		Regulation of antibiotic use in human health	When antibiotics are effectively regulated, it contributes to reduced misuse	WHO country situation analysis; data in literature
		Existence of surveillance program for AMR epidemiology and antibiotic use	Surveillance is a key component to adapt guidelines and guide action on AMR	WHO country situation analysis; data in the literature
		Antibiotic stewardship programs in hospitals and community	Antibiotic stewardship improves the appropriate use of antibiotics	Few data from literature
		National public awareness campaign	Informed citizens are more likely to use resources wisely	Estimates from literature
		Regulation of antibiotic promotion	Promotional practices can drive overuse	WHO country situation analysis
		Financial support for the development of new antibiotics	Incentives for new drugs may create new technologies	Various data in literature

Abbreviations: AMR, antimicrobial resistance; CDC, US Centers for Disease Control and Prevention; DPSIR, Driver-Pressure-State-Impact-Response; ECDC, European Centre for Disease Prevention and Control; FAO, Food and Agriculture Organization of the United Nations; IHME, Institute for Health Metrics and Evaluation; UN, United Nations; UNICEF, United Nations Children's Fund; WHO, World Health Organization.

**Table 2:** State of AMR: Examples of relevant resistant pathogens in human health.

Category of pathogens	Pathogen	Epidemiological features	Main current resistance problem
Gram-negative bacteria	*Acinetobacter* spp.**/‡‡‡	Mostly a nosocomial pathogen causing pneumonia and bacteremia	Carbapenems
	*Campylobacter* spp.**/‡‡	Community pathogen and leading cause of acute diarrhea worldwide	Fluoroquinolones
	*Escherichia coli* (ESBL**/‡‡‡ and CRE***/ ‡‡‡)	Frequent cause of bloodstream and urinary tract infection in both healthcare- and community-acquired infection. Frequent foodborne pathogen	Cephalosporins (ESBL) and carbapenems (CRE)
	*Helicobacter pylori*‡‡	Community pathogen causing gastrointestinal infection (gastritis)	Macrolides
	*Hemophilus influenzae*‡	Community pathogen causing pneumonia, epiglottitis and bacteremia, and meningitis in infants and young children	Ampicillin
	*K*. *pneumoniae* (ESBL**/‡‡‡ and CRE***/ ‡‡‡)	Severe hospital- and community-acquired infections. Responsible for urinary, respiratory and bloodstream infections	Cephalosporins (ESBL) and carbapenems (CRE)
	Neisseria gonorrhoeae***/‡‡	Community acquired STD resulting in infections of the genitals and pharyngitis	Extended-spectrum cephalosporins
	Pseudomonas aeruginosa**/‡‡‡	Nosocomial opportunist but also present in the community/major cause of pneumonia, bacteremia, and urinary tract infections	Numerous drugs, including last-choice antibiotics
	*Salmonella* spp.**/‡‡	Foodborne pathogen in the community. More prevalent in LMICs	Fluoroquinolones
	*Shigella* spp.‡	Foodborne pathogen in the community. More prevalent in LMICs	Fluoroquinolones
Gram-positive bacteria	Clostridium difficile***	Antibiotic-associated diarrhea and colitis	MDR strains
	*Enterococcus* spp.**/‡‡	Nosocomial infections in immunocompromised patients. Frequent agent of endocarditis	Vancomycin
	*Staphylococcus aureus* (MRSA**/‡‡, VRSA*/‡‡)	Leading healthcare and community pathogen that can cause severe infections	MDR to beta-lactams, vancomycin, and many other antibiotic classes
	Streptococcus pneumoniae*/‡	Upper respiratory tract infections. Most common cause of pneumonia worldwide	Combined penicillins and macrolides
Other bacteria	Mycobacterium tuberculosis**	Primarily an infection of the respiratory system. Common infectious cause of death in LMICs	XDR strains (resistance to any drug is possible)
Other pathogens	*Candida* spp. (fungus)**	Many infections from skin to bloodstream infections possibly affecting immunocompromised patients	Fluconazole and MDR strains (e.g., *Candida auris*)
	*Plasmodium falciparum* (parasite)	Community infection, especially in the tropical belt including sub-Saharan Africa and Southeast Asia	Artemisinin

The table has been compiled using data from the US Centers for Disease Control and Prevention (CDC), the European Centre for Disease Prevention and Control, WHO, and the Center for Disease Dynamics, Economics and Policy. To give a sense of priority, a reference is made to the US CDC classification of “Antibiotic resistance threats in the United States, 2013” using asterisks (*, **, ***; see footnotes below) [26] but prioritization of pathogens may differ according to countries and regions. An additional reference is made to the WHO “Global priority list of antibiotic-resistant bacteria to guide research, discovery, and development of new antibiotic” using double-dagger signs (‡, ‡‡, ‡‡‡; see footnotes below) [8]. Abbreviations: CRE, carbapenem-resistant Enterobacteriaceae; ESBL, extended spectrum beta-lactamase; LMICs, low- and middle-income countries; MDR, multidrug resistant; spp., species; STD, sexually transmitted disease; XDR, extremely drug resistant.

* Concerning threats

** serious threats

*** urgent threat [26].

‡ Medium

‡‡ high

‡‡‡ critical [8].

**Table 3:** Outcomes of AMR: Potential measurements of impact.

Impact	Indicators
Health impact	Morbidity (increased complications): admission to intensive care and incidence of *C*. *difficile* infection in hospitalized patients
	Mortality: attributable mortality from blood and CSF isolates for selected pathogens
Economic impact	Extra healthcare costs: diagnostics, use of second-line drugs, increase of time in care, and prolonged hospital stay
	Indirect costs such as loss of productivity and costs of not doing interventions because of AMR
	Societal costs to address the problem of AMR: costs of surveillance, conservation programs, and support for R&D
	Loss of productivity in animal health
Societal impact	Lack of trust in the healthcare system, fear of medical procedures, and barriers to poverty eradication

Abbreviations: AMR, antimicrobial resistance; CSF, cerebrospinal fluid; R&D, research and development.

The fourth challenge is data. Developing surveillance capacities is one of the core legal requirements of the International Health Regulations [27]. However, many countries have so far failed to meet this requirement. Multilateral initiatives such as the global health security agenda, a partnership of over 50 countries, can support the capacity to collect data in LMICs [28]. In addition, as surveillance programs for some specific diseases such as tuberculosis have already achieved significant coverage [29], synergies can be exploited to expand AMR surveillance. A global sentinel point prevalence study would also fill important epidemiological data gaps in LMICs. Moreover, citizen science projects have a potential for collecting more data on AMR, particularly regarding levels of resistance in the normal microbial flora [30]. Finally, the use of the DPSIR framework will help generate important new data on the regulatory component, as evaluation of this component is currently lacking. This in turn will aid the development of appropriate policies against AMR for the many countries that currently lack them.

The fifth challenge is reporting. Methodological issues should be addressed transparently, and results should be presented to avoid conveying simplistic and misleading policy messages. To ensure transparency, a data platform is needed in which information about each country will be collected and synthesized. A dashboard of indicators is likely to be necessary for a problem as complex as AMR. Because monitoring aims at informing policy, special attention must be devoted to communicating the findings online to make the most of data visualization for each country. Indicators—for example, those tracking countries’ actions against AMR—can be associated with appropriate visualization methods (e.g., traffic lights) to reflect countries’ responses so far. To increase the benefit for policy makers, countries with high or rapidly rising AMR levels will require further investigation and analysis, such as in-depth qualitative case studies that shed light on why particular AMR interventions do or do not succeed.

## Next steps

Fostering a global transformation to deal with AMR [31] requires political commitment and relevant governance mechanisms [32]. An effective mechanism to curb AMR globally would be the adoption of binding targets limiting antimicrobial use [10]. While it may take a long time for states to adopt these targets, expanding monitoring of AMR control will reduce information gaps and help governments maintain their commitments to tackle the problem. WHO, in collaboration with other relevant international organizations including the World Organization for Animal Health (OIE), the Food and Agriculture Organization (FAO), and the recently created UN Interagency Coordination Group on AMR should lead the process internationally.

As the first step is to define the scope of AMR monitoring, the DPSIR framework provides an integrated approach that matches the complex nature of the problem. The creation of an independent monitoring mechanism coordinated by WHO will be the next step. The recent difficulties that have hobbled the implementation of the International Health Regulations underscore the limits of countries’ self-assessment [33], but the work conducted by the Strategic Advisory Group of Experts on Immunization demonstrates that independent monitoring is possible within the remit of the organization [34]. Since expanded monitoring will help identify countries most in need of support, it should be linked to an international funding mechanism to support conservation efforts in LMICs [35].

A robust approach to expand AMR monitoring demands a wide participation of global health actors. The recent launch of the “Conscience of Antimicrobial Resistance Accountability” (CARA), which aims to monitor the state of national responses, is a step in this direction [36]. Drawing from environmental governance, in which civil society has been playing a recognized role in collecting and evaluating policy responses by public actors [37], an international legal mechanism empowering civil society to participate in monitoring could further serve as a model for AMR governance [38]. As many high-income countries already collect AMR data on a yearly basis, academic institutions could use the DPSIR framework to develop case studies about these countries. Finally, financial support from countries and donors at the forefront of the fight against AMR is needed to strengthen WHO, the work of which has been hampered by unpredictable funding, leading (inter alia) to counterproductive internal competition. In line with the broad mandate conferred by its constitution and the recent UN political declaration [3], strong leadership by WHO is critical to orchestrate the expansion of AMR monitoring and contribute to successful AMR governance.

## Abbreviations

AMR: antimicrobial resistance
CARA: Conscience of Antimicrobial Resistance Accountability
CDC: US Centers for Disease Control and Prevention
CRE: carbapenem-resistant Enterobacteriaceae
CSF: cerebrospinal fluid
DPSIR: Driver-Pressure-State-Impact-Response
ECDC: European Centre for Disease Prevention and Control
ESBL: extended spectrum beta-lactamase
FAO: Food and Agriculture Organization
GAP: Global Action Plan
IHME: Institute for Health Metrics and Evaluation
LMICs: low- and middle-income countries
MDR: multidrug resistant
OIE: World Organization for Animal Health
R&D: research and development
spp.: species
STD: sexually transmitted disease
UNICEF: United Nations Children's Fund
WHO: World Health Organization
XDR: extremely drug resistant
